# Biodegradation of Indanthrene Blue RS dye in immobilized continuous upflow packed bed bioreactor using corncob biochar

**DOI:** 10.1038/s41598-021-92889-3

**Published:** 2021-06-28

**Authors:** Swati Sambita Mohanty, Arvind Kumar

**Affiliations:** Department of Chemical Engineering, National Institute of Technology Rourkela, Rourkela, Odisha 769008 India

**Keywords:** Biological techniques, Environmental sciences

## Abstract

The current study describes the aerobic biodegradation of Indanthrene Blue RS dye by a microbial consortium immobilized on corn-cob biochar in a continuous up-flow packed bed bioreactor. The adsorption experiments were performed without microbes to monitor the adsorption effects on initial dye decolorization efficiency. The batch experiments were carried out to estimate the process parameters, and the optimal values of pH, temperature, and inoculum volume were identified as 10.0, 30 °C, and 3.0 × 10^6^ CFU mL^−1^, respectively. During the continuous operation, the effect of flow rate, initial substrate concentration, inlet loading rate of Indanthrene Blue RS on the elimination capacity, and its removal efficiency in the bioreactor was studied. The continuous up-flow packed bed bioreactor was performed at different flow rates (0.25 to 1.25 L h^−1^) under the optimal parameters. The maximum removal efficiency of 90% was observed, with the loading rate varying between 100 and 300 mg L^−1^ day^−1^. The up-flow packed bed bioreactor used for this study was extremely useful in eliminating Indanthrene Blue RS dye using both the biosorption and biodegradation process. Therefore, it is a potential treatment strategy for detoxifying textile wastewater containing anthraquinone-based dyes.

## Introduction

Rapid urbanization increases the demand for fast industrialization, thereby causing problems for the environment. The release of immense amounts of wastewater from the industrial sector decreases freshwater availability^1,2^, thus leading to pollution issues worldwide if not appropriately treated. Industries such as textile, printing, tannery, paint, plastic, cosmetics enormously used synthetic dyes because of their intense and solid colors. O’Neill et al. 1999 stated the recognizable amount of colors in water to be higher than 1 mg L^−1^^3^. Generally, 100.000 synthetic dyes and dyestuffs are utilized by dyeing industries^4^, and nearly 10–15% of the unfixed dyes are released to the water stream directly without being treated. The entry of untreated textile effluents into the water bodies alters the pH, BOD, COD and reduces the sunlight penetration^5^, and thereby disturbs the biodiversity of that system.

Based on the classification of dyes, their disposal into the water stream ranges from 2% (basic dyes) to 50% (reactive dyes) of the initial dye concentration^3,6^. Among these reactive dyes, azo and anthraquinone dyes represent a significant group of synthetic dyes^7^. Their complex structure makes them highly toxic and resists degradation^8,9^. Anthraquinone dyes are among the major groups of colored pollutants highly resistant to degradation due to the presence of the fused aromatic rings, which help them to remain colored for a longer time^10–12^. They represent about 15% of total dyes are water-insoluble due to the existence of the chromophoric group designed by the conjugation of C=O and C=C^13,14^. Among the anthraquinone dyes, “Indanthrene Blue RS,” commonly used for cotton and silk dyeing, is primarily observed in the wastewater of textile industries.

Various physicochemical methodologies such as adsorption, oxidation, coagulation, precipitation, and membrane filtration^15^ employed for decolorization of textile effluents are cost-intensive, less efficient, and result in large aggregates of secondary pollutants^16,17^. Therefore, there is a burgeoning demand to establish cost-effective methods to decolorize these polluting dyes. Biological processes using bacteria, algae, yeast, and fungi are excellent alternative methods compared to physicochemical methods in decolorizing textile effluents. They have less operational cost, are eco-friendly, and under optimal operating conditions, produce less sludge compared to other methodologies^18,19^. This biodegradation process has become a promising method as it completely decolorizes the dye and transforms them into a non-toxic chemical form^20,21^. Under optimized, aerobic or anaerobic conditions using microorganisms, a significantly high percentage of decolorization and degradation of dyes can be achieved^22–24^.

The bioremediation of textile wastewaters is continuously expanding within environmental biotechnology as it is fast and efficient. Pure bacterial strains generally are incapable of degrading the dyes completely, producing carcinogenic aromatic amines as intermediates, which further need to be decomposed^25^. It’s crucial to scale up and maintain pure cultures for wastewater treatment systems in large-scale^26^. In the recent past, many consortiums having enhanced degradation abilities have been studied. The microbial consortium has been used over pure cultures for dye degradation as it possesses a high degree of mineralization and biodegradation^7^ due to the synergistic interaction in the metabolism of the bacterial community. In a consortium, the bacterial strains metabolize the molecular structure of dye by attacking at various positions of the aromatic rings or by using metabolites formed by the dominant strains to degrade further^27,28^. Nowadays, trials on bacterial-bacterial synergism are being utilized to develop new environmentally-friendly abatement technologies to degrade the textile dye wastes without producing toxic metabolites.

Bioreactors are the basis of all biotransformation processes, such as the production of vaccines, enzymes, nutrients, etc., and biodegradation activities^29^. Various biodegradation assays of dye were performed using reactors in batch mode, but continuous reactors are recognized as more efficient and appropriate for real-time applications^30^. Simultaneous biosorption processes and biodegradation (BB) were established to remove high concentration dyes involving adsorption followed by biological treatment. Using this advanced treatment strategy effectively removes the contaminants converted into harmless compounds in the bioremediation process. The BB process is an economically feasible, highly effective, and environmentally friendly process and has advantages in practical applications compared to other conventional techniques^31^. The efficiency of a continuous reactor can be increased under optimal conditions by using packing materials^32,33^. A continuous method for effluent treatment needs to be developed by considering the large-scale elimination of anthraquinone dyes, mainly from the textile industry, into the waterways. Continuous mode bioreactor operations for effluent degradation under aerobic conditions require immobilized biofilms suspended to biological systems. Biofilms can be developed by encapsulating the microorganisms into the packing material, which supports growth. This immobilization system has been reported to be better than the free cell system as it offers a high loading rate and inhibits biomass washout. The other advantages of using this technology include higher stability, reduced land usage, lower operational cost, and waste recycling^34^. Therefore, it is difficult to find efficient treatment technologies for the dyeing industries to meet the environmental sustainability requirement. Immobilized cell technology has become an accepted method for treating dye wastewaters and thereby has achieved increasing attention. Studies on dye biodegradation have been reported to be primarily done in batch reactors.

Moreover, continuous reactors are effective and appropriate for real-time operation^30^. The continuous reactor efficiency could be improved by optimal operating conditions in packed bed mode^32^. Over several years, activated carbon was used for wastewater treatment. It provides a support matrix for biofilm development and a large surface area to adsorb substances passing through the reactor^35,36^.

Pyrolyzed biomass, commonly referred to as biochar, is an alternative to activated carbon, which is explored as a low-cost strategy for treating wastewater. Biochar is a carbon-rich compound synthesized by the breakdown of various organic waste materials^37^. It has been observed that a wide variety of agricultural waste, such as wheat straw, bamboo, rice straw, coffee husk, and coconut shell can be used as biomass feedstock for biochar generation^13,38,39^. Due to the abundant production and low expenses of these wastes, converting them into biochar contributes significantly to environmental sustainability and increases the economic viability of their wide applications. The advantages of biochar in dye treatment techniques have already been validated^30,40^. Initially, mass transfer of contaminants from an aqueous solution to the surface of an immobilized carrier was expedited using biochar adsorption^39,41^. Finally, when active microbes were immobilized with biochar, the immobilized microbial multiplication was boosted, significantly increasing the elimination of pollutants^42^. Biochar, compared to traditional carrier materials are more effectively reduces the stress of adverse environmental conditions and enhances the activities of microorganisms^43^. As a result, it is suggested that biochar will considerably increase the practical viability of microbial immobilization for treating textile wastewater.

Corn-cob (CC) biochar has been identified as a suitable matrix for immobilizing the bacterial cells in the continuous flow bioreactor. It facilitates the adsorption of a large number of bacterial cells and biofilm formations, both inside and on the surface of the biochar^37^. To date, no work has been reported on the aerobic bioremediation of Indanthrene Blue RS using CC-biochar in continuous up-flow packed-bed bioreactors. In the present study, Indanthrene Blue RS dye biodegradation was performed by immobilizing cells on CC-biochar. In an up-flow packed bed bioreactor (UFPBBR), continuous studies were performed to evaluate its effect on biodegradation using consortium-BP. Process parameters, including initial dye concentration, inoculum volume, and flow rate, were optimized for maximum degradation efficacy. The performance of the reactor efficiency was evaluated with immobilized cells operated in batch and continuous mode. The UV–vis spectrophotometer was used to measure the percentage of decolorization. Cells immobilized on CC-biochar continuously removed a high concentration of Indanthrene Blue RS (500 mg L^−1^) by using the BB process. The findings suggest that this BB process is more effective than conventional treatment approaches and has higher effectiveness.

## Materials and methods

### Dyes and chemicals

The textile anthraquinone vat dye, Indanthrene Blue RS, was procured from Sigma-Aldrich, India. The composition of liquid mineral‐base medium (MBM) includes NaNO_3_ (0.3%), KCl (0.05%), K_2_HPO_4_ (0.1%), MgSO_4_ (0.05%), and yeast extract (0.02%) with glucose (1%). The alkaline pH of the media was maintained by using sterilized NaOH (1 M). The chemicals and media components used in these experiments were of analytical grade.

### Microorganisms and culture conditions

The isolation of a pure culture of *Bacillus flexus* TS8, *Proteus mirabilis* PMS, and *Pseudomonas aeruginosa* NCH were carried out from the textile wastewater under laboratory conditions. Under aerobic conditions, the culture was grown in a 250 mL flask, each containing 100 mL of nutrient broth, and was incubated at 30 °C for 24 h. The consortium-BP was prepared by aseptically transferring 24 h grown culture (50 mL) of each strain into 250 mL flasks to retain the equal number of cells in both the pure culture and the consortium, respectively.

### Evaluation of corn-cob biochar as a packing material

The corn-cob was pyrolyzed at 400 °C to prepare biochar and was used as a supporting material for microbial immobilization. The biochar was sterilized before use by autoclaving for 15 min at 15 psi (121 °C) to ensure that microbes were not involved during the adsorption process. FT-IR analysis by KBr pellet technique was performed to assess the presence of functional groups in biochar before and after adsorption using the Thermofisher Scientific, Nicolet IS10 in ATR mode (with the 16-scan speed in the mid-IR range of 400–4000 cm^−1^). CHNS analysis was carried out to determine the composition of existing elements in biochar, such as carbon, hydrogen, nitrogen, and sulfur (Elementar Analysen Systeme, Germany/Vario EL). SEM analysis (JEOL JSM-6084LV) determines the surface morphology of the biochar. Biochar porosity, surface area, and adsorption volume were evaluated using a BET analyzer (Quanta chrome/AUTOSORB-1).

The corn-cob biochar (CC-biochar) has also been examined for the adsorption and desorption of the Indanthrene Blue RS dye packed in the column. For adsorption experiments, 2.5 g of CC-biochar was added with Indanthrene Blue RS (100 mg L^−1^) to 100 mL of MBM. The flasks were incubated in an isothermal shaker (120 rpm) at room temperature. The samples were withdrawn at a regular time interval (30 min) for 4 h. The supernatant was then centrifuged at 8000 rpm for 10 min. The percentage of dye removal was measured spectrophotometrically at 520 nm. The desorption experiments were carried out by subsequently drying and transferring the spent CC-biochar sample into NaCl solution (100 mL) (Voudrias et al.^44^). The flasks were incubated in an isothermal shaker, and the study was carried out as specified in adsorption experiments. The final concentrations of desorbed dye were determined spectrophotometrically at 520 nm.

### Adsorption study

In Erlenmeyer flasks of 250 mL, this study was conducted by subsequently changing the initial dye concentrations (25 to 150 mg L^−1^), contact time (5 to 240 min), adsorbent dose (0.2 to 2 g L^−1^), and temperature (25 to 50 °C). A UV–vis spectrophotometer was used to measure the dye concentration in the supernatant at 520 nm. The quantity of dye adsorbed by the unit weight of CC-biochar at time *t*, ${q}_{t}$(mg/g) and the percentage of removal of dye, R was determined as follows:

1 $${q}_{t}\,=\,\frac{\left({C}_{0}-{C}_{t}\right)\,\times\,V}{W}$$

2 $$R\%=\frac{\left({C}_{0}-{C}_{t}\right)}{{C}_{0}}\times 100$$

where ${C}_{0}$ and ${C}_{t}$ (mg L^−1^) represents dye concentrations at the initial and any time (t, min), V, ${q}_{t}$, and W represents the volume of the solution (L), the adsorbed amount (mg g^−1^) at any time (t, min), and the mass of adsorbent (g), respectively.

#### Adsorption isotherms

The adsorption isotherms were determined by adding 1 g L^−1^ of CC-biochar in 100 mL dye solution with varying concentrations (25, 50, 100, and 150 mg L^−1^). pH 10.0 was maintained, and the experiment was performed for 240 min with continuous shaking at 30 °C to validate the equilibrium time. After 90 min incubation, the solution reached equilibrium. The difference between the two concentrations determined the quantity of adsorbed dye (mg g^−1^) on the surface of the adsorbent.

The Freundlich and Langmuir isothermic equations were used to evaluate the experimental equilibrium data for Indanthrene Blue RS adsorption. The Langmuir isotherm model is established on the presumption that the adsorbent has monolayer coverage on the outer surface of the adsorbent can be defined linearly as:

3 $$\frac{1}{{q}_{e}}=\frac{1}{{q}_{m}}+\frac{1}{{K}_{L}{q}_{m}{C}_{e}}$$

where ${K}_{L}$ is the adsorption energy-related Langmuir constants, ${q}_{m}$ is the maximum adsorption capacity. The linear plot of $1/{q}_{e}$ Vs. $1/{C}_{e}$ can be used to derive the values of ${K}_{L}$ and ${q}_{m}$.

The Freundlich isotherm refers to heterogeneous adsorption surfaces. The following equation represents the linear form of freundlich isotherm as:

4 $$\mathrm{ln}{q}_{e}=\mathrm{ln}{K}_{F}+\frac{1}{n}\mathrm{ln}{C}_{e}$$

where ${K}_{F}$ (mg g^−1^) is the Freundlich isotherm constant related to the adsorbent’s adsorption capacity, n is the Freundlich isotherm constant associated with the adsorbent affinity.

#### Adsorption kinetics

The Indanthrene Blue RS adsorption on the surface of the CC-biochar was evaluated using the basic kinetic models to study the adsorption process. In this study, kinetic models of the pseudo-first-order and pseudo-second-order are evaluated to identify the best fit model for the observed data.

##### Pseudo-first-order kinetic model

The kinetic model of the pseudo-first-order equation stated that over time, the solute adsorption rate was related to the variation in the concentration of saturation and the adsorbed quantity. In most situations, the adsorption mechanism followed by diffusion over a boundary obeys the pseudo-first-order kinetic rate equation. The linear relation between the adsorbed dye ${q}_{t}$ (mg/g) and time t is described as:

5 $$\mathrm{ln}\left({q}_{e}-{q}_{t}\right)=\mathrm{ln}{q}_{e}-{k}_{1}t$$

The pseudo-first-order kinetic rate constants ${q}_{e}$ (mg g^−1^) and ${k}_{1}$ (min^−1^) are calculated from the intercept and the slope of the graph obtained from $\mathrm{ln}\left({q}_{e}-{q}_{t}\right)$ vs. t.

##### Pseudo-second-order kinetic model

The kinetic model of the pseudo-second-order equation fits the adsorption mechanism, with chemisorptions becoming the rate-control. The pseudo-second-order model may define the linear form of adsorption kinetics as:

6 $$\frac{t}{q}=\frac{1}{{K}_{2}{{q}_{e}}^{2}}+\frac{1}{{q}_{e}}t$$

The $t/{q}_{t}$ vs. t plot provides a straight line with $1/{q}_{e}$ as slope and $1/{K}_{2}{q}_{e}$ as intercept. The ${K}_{2}$ value is computed from the intercept slope using ${q}_{e}$ estimated from the slope.

### Microbial growth and degradation of Indanthrene Blue RS in immobilized cell batch experiments

The microbial growth was obtained by inoculating consortium-BP in the MBM medium. The degradation studies were carried out by adding a 5% (v/v) inoculum volume to various Indanthrene Blue RS concentrations. An aliquot (2 mL) was withdrawn at a regular interval and was harvested by centrifuging at 8000 rpm for 10 min for separation of cell biomass. The decolorization efficiency was measured spectrophotometrically by observing the culture supernatant at 520 nm (Shimadzu, UV-1800). The percentage of decolorization was defined as:

7 $$\%Decolorization\,=\,\frac{(Initial\,absorbance\,-\,Final\,absorbance)}{Initial\,Absorbance}\,\times\,100$$

### Parameter optimization

Batch studies were carried out to optimize the process parameters such as pH (6.0 to 12.0), temperature (20 to 40 °C), and inoculum volume (1.0 × 10^6^ to 5.0 × 10^6^ CFU mL^−1^). The parameters were varied at a fixed concentration of Indanthrene Blue RS (200 mg L^−1^). All the studies were performed in triplicates.

### Effect of dye concentration in the immobilized cell batch reactor

Indanthrene Blue RS dye decolorization was performed at varying dye concentrations using immobilized consortium cells in batch bioreactors at the optimal process parameters. The CC-biochar was used as the immobilization media. Such studies were performed to determine the maximum tolerated level up to which the microbes can actively degrade the dye without inhibition of the substrate and other possible toxic effects. The Indanthrene Blue RS dye decolorization studies were performed at concentrations ranging from 100 to 700 mg L^−1^.

### Up-flow packed bed bioreactor set up and operation

**Figure 1:**
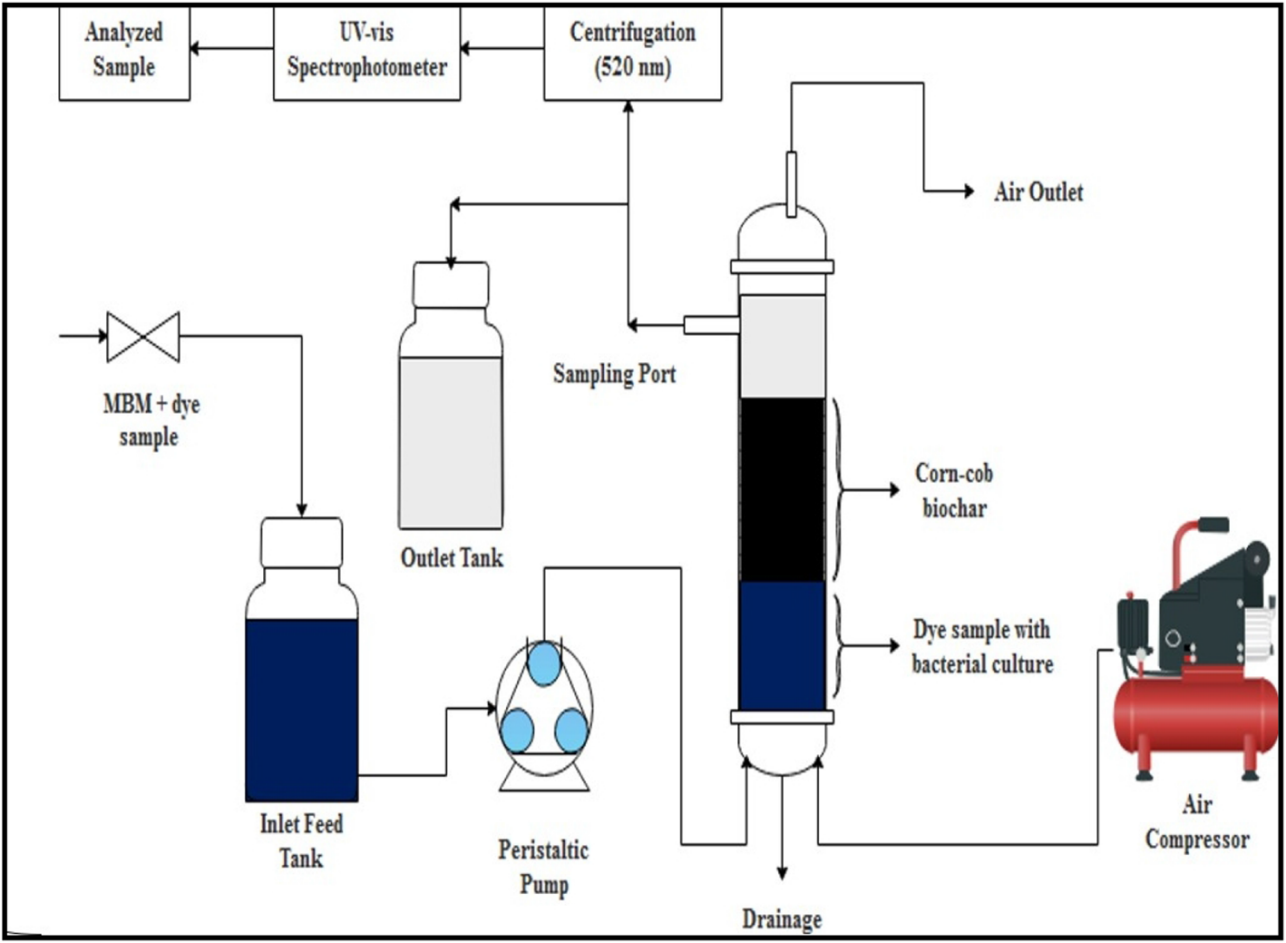
The schematic diagram for up-flow packed bed bioreactor in continuous mode.

An up-flow packed bed bioreactor (PBBR) has been designed with a flat base using Perspex glass (Fig. 1) with 60 cm long and 8 cm in diameter, respectively. The PBBR consists of an inlet feed tank, peristaltic pump, air compressor, outlet tank, CC-biochar, and a reactor with a sampling port. The PBBR was operated under aerobic conditions by providing purified air in the bed. The biochar was packed across two tube grooves up to a height of 20 cm and was covered at the base by sieves (metal) of < 0.5 μm size and cotton (saturated with dye). The bioreactor was loaded with 400 g of CC-biochar with 3016 mL and 1005 mL of the total volume and working volume, respectively. The microbial culture in the liquid mineral-base medium was circulated to immobilize the bed adequately with consortium cells through the peristaltic pump. The medium was supplied to the bioreactor upward to prevent channeling effects and increased retention time. The airflow rate of 0.1 LPM was maintained and supplied by an air compressor at 30 ± 3 °C (room temperature).

The bioreactor was subsequently operated with 500 mg L^−1^ of Indanthrene Blue RS dye solution in continuous mode from a feed tank of 25 L. The varying feed rate (0.25 to 1.25 L h^–1^) was maintained for proper distribution using a peristaltic pump. The bioreactor inoculum was 10% of the volume having 3.0 × 10^6^ CFU/mL. The optimal parameters achieved from the batch experiments were considered for the bioreactor operation. The Indanthrene Blue RS decolorization was performed over 20 days. The samples were withdrawn on complete decolorization, centrifuged at 7168×*g* for 10 min, and quantified using UV–vis spectroscopy.

### Degradation analysis and performance equations

After complete decolorization, the supernatant was separated by centrifuging 50 mL of the culture medium at 7168×*g* for 10 min. The extent of decolorization was determined at 520 nm using a UV–vis spectrophotometer. The PBBR performance in the continuous mode was calculated under optimal conditions and at varying inlet loading rates in the form of elimination capacity (EC) and removal efficiency (RE) and defined by:

8 $$\%\,Removal\,Efficiency\left(RE\right)=\frac{{C}_{in}-{C}_{out}}{{C}_{in}}$$

9 $$Elimination\,Capacity\left(EC\right)=Q\frac{{C}_{in}-{C}_{out}}{V}$$

10 $$Inlet\,Loading\,Rate\left(ILR\right)=\frac{{C}_{in}}{V}Q$$

where ${C}_{in}$ and ${C}_{out}$ represents the inlet and outlet concentrations of Indanthrene Blue RS. Q, V represents the volumetric feeding flow rate and the working volume, respectively.

## Results and discussion

### Biochar characterization and analysis

The efficiency of biochar’s sorption depends mainly on the chemical reactivity and functionality of the biochar surface and its porosity. Therefore, a detailed understanding of the functional groups on the biochar surface allows for a better interpretation of the sorption process. When the biomass is heated to 350 to 650 °C, the chemical bonds break and rearrange, forming new functional groups^41^. The FT-IR analysis of the biochar indicates the participation of the stretching vibration of C–C (quinones) and N–H (amine) at 1101.1 cm^−1^ and 3471.2 cm^−1^, respectively, correspond to the N–H (amine) bending vibration at 1602.6 cm^−1^ before biosorption^45,46^ (Supplementary Fig. 1). The FT-IR spectrum shows that biochar is represented by oxygenated hydrocarbons functional groups, reflecting the carbohydrate composition of cellulose and hemicelluloses^46^. The presence of the functional group after sorption results in the stretching vibrations of =C–O (ketones, aldehydes, and esters), C–N (nitrile), C–H (alkyl), O–H (alcoholic and phenolic), and N–H (amine) at 1051.0 and 1708.6 cm^−1^, 1261.2 cm^−1^, 1386.5 cm^−1^, 3235.9 cm^−1^, and 3398.1 cm^−1^, respectively, correspond to the bending vibrations of –C–H (alkyl), N–H (amine) at 1386.5 cm^−1^ and 1579.4 cm^−1^ (Supplementary Fig. 1). The FT-IR spectrum exhibited the appearance or shifting of few peaks after the adsorption of Indanthrene Blue RS dye on the CC-Biochar.

The spectral analysis of CHNS (after sorption) resulted in an increase in the significant components of the respective samples: Carbon (70.97%), Hydrogen (1.22%), and Nitrogen (14.96%), and thus indicating its adsorption. On increasing the pyrolysis temperature, there is an increase in the carbon content of the biochar^38,47^. The high carbon content of the biochar possibly suggests that it still contains a specific quantity of organic plant residues from plants (cellulose)^48^. Supplementary Table 1 illustrates the elemental analysis of corn-cob biochar before and after treatment.

The surface morphology of the CC-biochar sample revealed an adequate number of free pores or adsorption sites on the surface using a scanning electron microscope (SEM) (Supplementary Fig. 2). All these pores represent the efficiency of dye biodegradation by microbial strain on the biochar surface. After pyrolysis, the morphology of CC-biochar was observed, and it revealed that biochar has a porous surface that facilitates bacterial cell growth and biofilm formation. SEM images captured at 20,000 × displayed the biofilm formation on the corn-cob biochar surface. Also, higher bacterial cell density was observed within the corn-cob biochar when thin biochar cross-sections were examined under SEM (Supplementary Fig. 3).

BET analysis concluded the surface area of 26.17 m^2^ g^−1^, an average pore diameter of 5.25 nm, and a total pore volume of 0.08 cm^3^ g^−1^, which was higher than that of the previous studies reported for biochar. The surface area is one of the most significant properties of biochar and depends on feedstock type^49,50^. Furthermore, the degradation of aliphatic alkyls and ester groups, and the exposure of the aromatic lignin core to higher pyrolysis temperatures, would result in an increased surface area^51^. The adsorption of organic contaminants into the biochar depends on the total volumes of the micropores and mesopores. The adsorption is further accelerated as the ionic radius is small, leading to the increased adsorption capacity of the biochar^39,43^. The biochar surface is generally negatively charged owing to the dissociation of functional groups containing oxygen, which causes electrostatic attraction between Biochar and positively charged molecules^39,52^. Rafiq et al. 2016 reported that an increase in temperature increased the biochar surface area^53^. The pore-blocking compounds are washed off or thermally damaged with rising pyrolysis temperature, increasing the surface area, which is readily accessible. As the temperature rises, the porosity of biochar increases because of lignin decomposition, the rapid production of H_2_ and CH_4_, and the aromatic condensation reaction^54,55^. Supplementary Fig. 4 presented the BET surface area plot for corn-cob biochar.

### Stand-alone adsorption studies of the packing material in batch mode

#### Effects of contact time and initial dye concentration, adsorbent dose, and temperature

**Figure 2:**
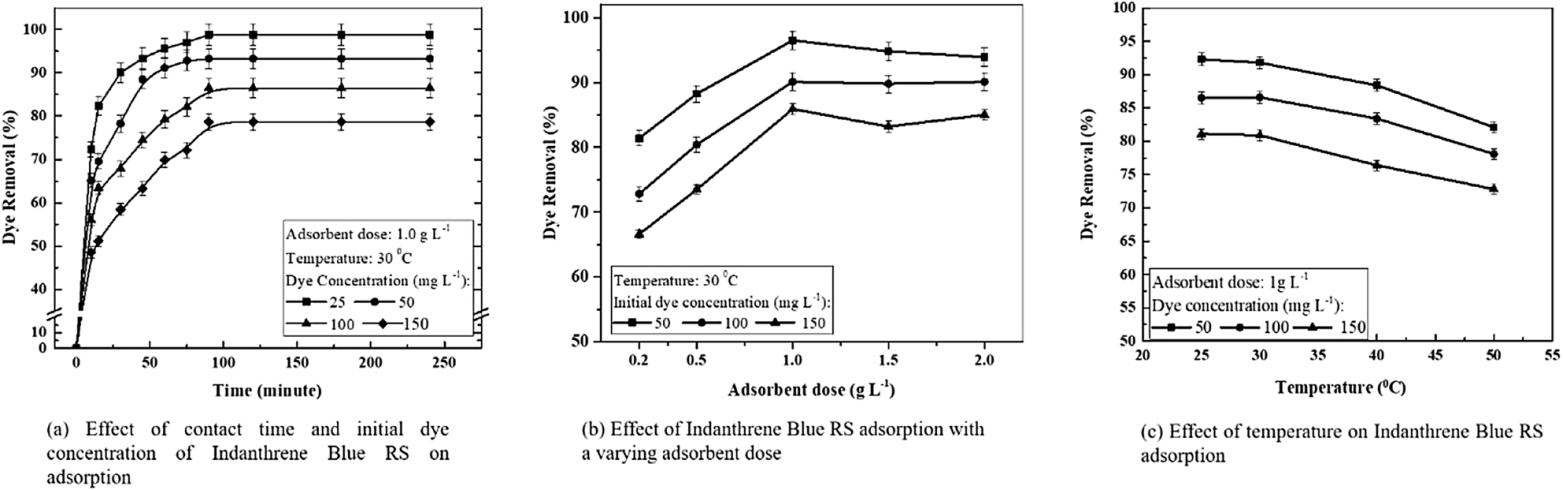
Effects of contact time and initial dye concentration, adsorbent dose, and temperature on Indanthrene Blue RS adsorption.

Before using the corn-cob biochar as the packing material, adsorption studies were performed without microbes to evaluate the adsorption efficiency in removing the dye. Indanthrene Blue RS dye adsorption studies were conducted at different concentrations using corn-cob biochar (Fig. 2a). At varying initial dye concentrations, an increase in the decolorization efficiency was observed with a fixed dose (1 g L^−1^) and temperature (30 °C). The study showed that equilibrium was attained within 90 min (1.5 h). Afterward, no significant changes in the degree of adsorption were found. Indanthrene Blue RS concentration decreases significantly as the incubation time increases. This finding suggested that the use of CC-biochar alone could partially remove Indanthrene Blue RS. More than 80% adsorption was measured for an initial concentration of up to 50 mg L^−1^, while the dye removal percentage was reduced to 78% and 69% for 100 and 150 mg L^−1^, respectively. In the above observation, it is apparent that the adsorption is very fast for the lower initial dye concentration. The adsorption capacity of corn-cob biochar for an initial dye concentration of 25, 50, 100, and 150 mg L^−1^ was found to be 7.28, 9.81, 4.78, and 2.22 mg g^−1^, respectively. The percent removal of dye reduces with a rise in initial concentration. This result was in agreement with that reported by Zheng et al., 2017^31^. It needs a longer time to achieve equilibrium, and with an increased concentration of dyes, competition for active adsorption sites will increase, and the adsorption process will slow down further^30,56^. It can be defined better by an adsorption mechanism where the dye molecules first enter a boundary layer, then diffuse to the adsorbent surface from the boundary layer film, and lastly diffuse through the adsorbent’s porous structure^38^. The other reason for this may be attributed to the water constraints such as cationic and anionic ions that significantly affected the adsorption capability of an adsorbent for effective dye removal^44,57,58^. Water containing cationic and anionic ions may compete with dye molecules for active sites on the adsorbent surface^58^. These competitions reduce dye adsorption capacities, causing it to become saturated quickly^59^.

Figure 2b shows the effect of adsorbent dose (ranging from 0.2 to 2.0 g L^−1^) on the dye adsorption capacity at three varying initial dye concentrations 50, 100, and 150 mg L^−1^. At first, the significant rise in adsorption with the increased dose can be due to the increased surface area and availability of more adsorption sites. After the critical dose of 1 g L^−1^, the adsorption rate is slowing increasingly. The percentage of dye removal is increased as there is rapid superficial adsorption on the adsorbent surface at a higher adsorbent dose, resulting in a lower concentration of the solute in the solution than when the adsorbent dose is low. Similarly, as the initial dye concentration for constant adsorbent dose increases, the adsorption of dye increases due to more dye molecules in the solution^60^.

The effect of temperature on the adsorption of Indanthrene Blue RS is presented in Fig. 2c. The studies were performed at three varying concentrations of dye (50, 100, and 150 mg L^−1^) and four different temperatures (25, 30, 40, and 50 °C) utilizing 1 g L^−1^ of adsorbent dose. It was observed that as the temperature rises from 25 to 30 °C, there was an increase in the adsorption rate. When the temperature rose from 35 to 50 °C, no significant difference in the removal percentage was observed^61^. Hence, the effect of temperature on Indanthrene Blue RS decolorization is negligible.

The above results, therefore, suggest that the use of corn-cob biochar as an adsorbent is not an effective method since the adsorbent will soon become saturated due to the low adsorbent capacity.

#### Adsorption kinetics and isotherms

**Figure 3:**
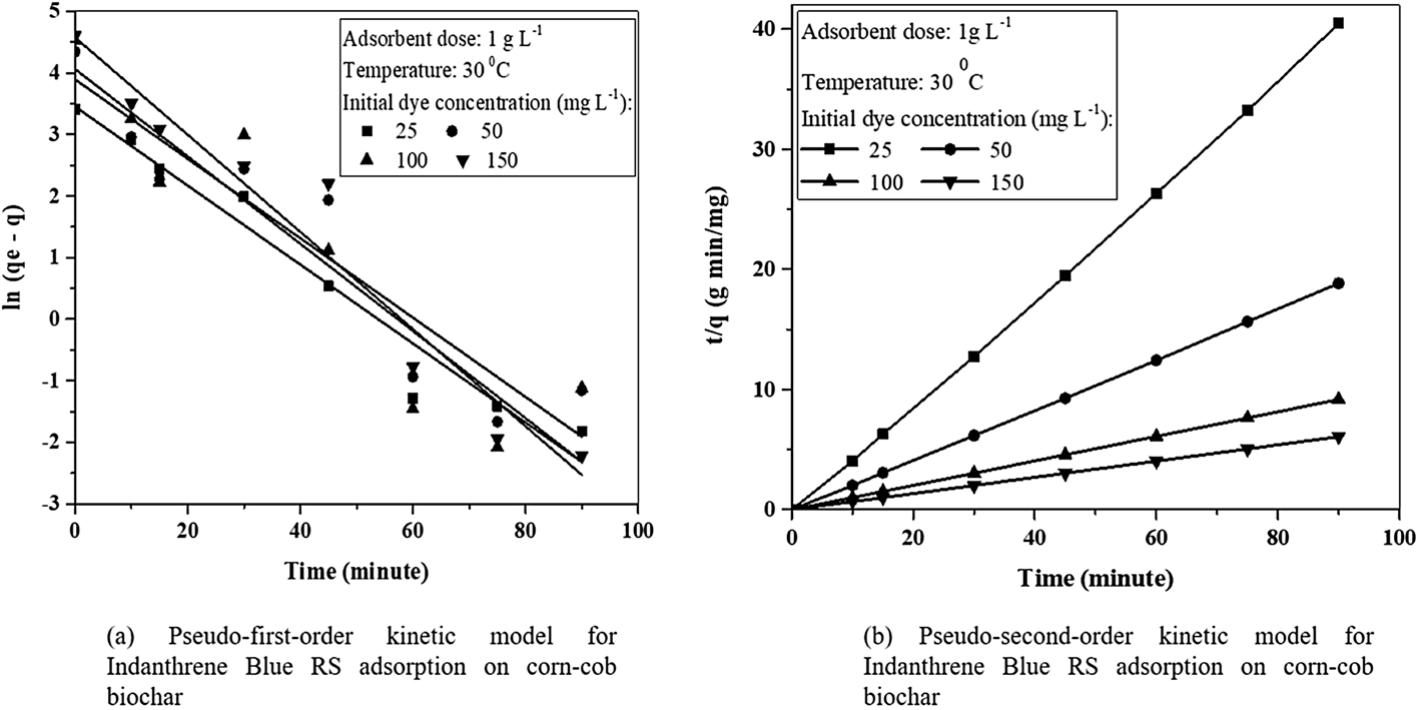
Kinetics for Indanthrene Blue RS adsorption.

The kinetic parameters were evaluated using linear plots of pseudo-first-order, pseudo-second-order kinetic models (Fig. 3a,b). The parameters were determined, as presented in Table 1. The plot of $ln\left({q}_{e}-{q}_{t}\right)$ vs. t will present a straight line with ${-k}_{1}$ and $ln{q}_{e}$ as slope and intercept, allowing to evaluate the adsorption rate constant ${k}_{1}$ and equilibrium adsorption capacity ${q}_{e}cal$ (Fig. 3a). It was found that the experimental data point does not fit a straight line and the values determined for ${k}_{1}$ and ${q}_{e}cal$ are given in Table 1. Hence, it can be inferred from the findings that the kinetics of Indanthrene Blue RS adsorption on corn-cob biochar is not likely to follow the pseudo-first-order kinetic model and thus not a diffusion-controlled phenomenon.

**Table 1:** Kinetic model parameters at 30 °C.

Indanthrene Blue RS concentration (mg L^−1^)	Pseudo-first order				Pseudo-second order		
	q_e,__exp_ (mg g^−1^)	q_e,__cal_ (mg g^−1^)	k_1_ (min^−1^)	R^2^	q_e,__cal_ (mg g^−1^)	k_2_ (g mg^−1^ min)	R^2^
25	7.28	4.68	0.08	0.95	7.41	0.67	1.00
50	9.81	5.79	0.07	0.87	9.81	0.69	1.00
100	4.78	1.48	0.06	0.88	4.78	0.55	0.99
150	2.22	0.14	0.06	0.96	2.22	0.45	0.99

The $t/{q}_{t}$ vs. t plot gives a straight line with $1/{q}_{e}$, and $1/\left({k}_{2}{{q}_{e}}^{2}\right)$ as slope and intercept are presented in Fig. 3b. The ${k}_{2}$ value is estimated from the intercept using ${q}_{e}cal$ value determined from the slope. Table 1 shows the estimated value of ${k}_{2}$, ${q}_{e}cal$, and their respective regression coefficient (R^2^) values. The R^2^ value is 1.00 for 25 and 50 mg L^−1^ and almost unity (0.99) for 100 and 150 mg L^−1^ of Indanthrene Blue RS, indicating that the Indanthrene Blue RS adsorption kinetics follow pseudo-second-order kinetic model. It may also be observed from Table 1 that ${q}_{e}cal$ values are very close to ${q}_{e}exp$ values that were obtained experimentally. Therefore, it can be stated that the pseudo-second-order kinetic model can describe the Indanthrene Blue RS adsorption on CC-biochar better than the pseudo-first-order kinetic model, and the mechanism is regulated by chemisorption. A similar result was observed by Zheng et al., 2017 for the adsorption of AO10 molecules onto MHSA-AC^31^.

**Figure 4:**
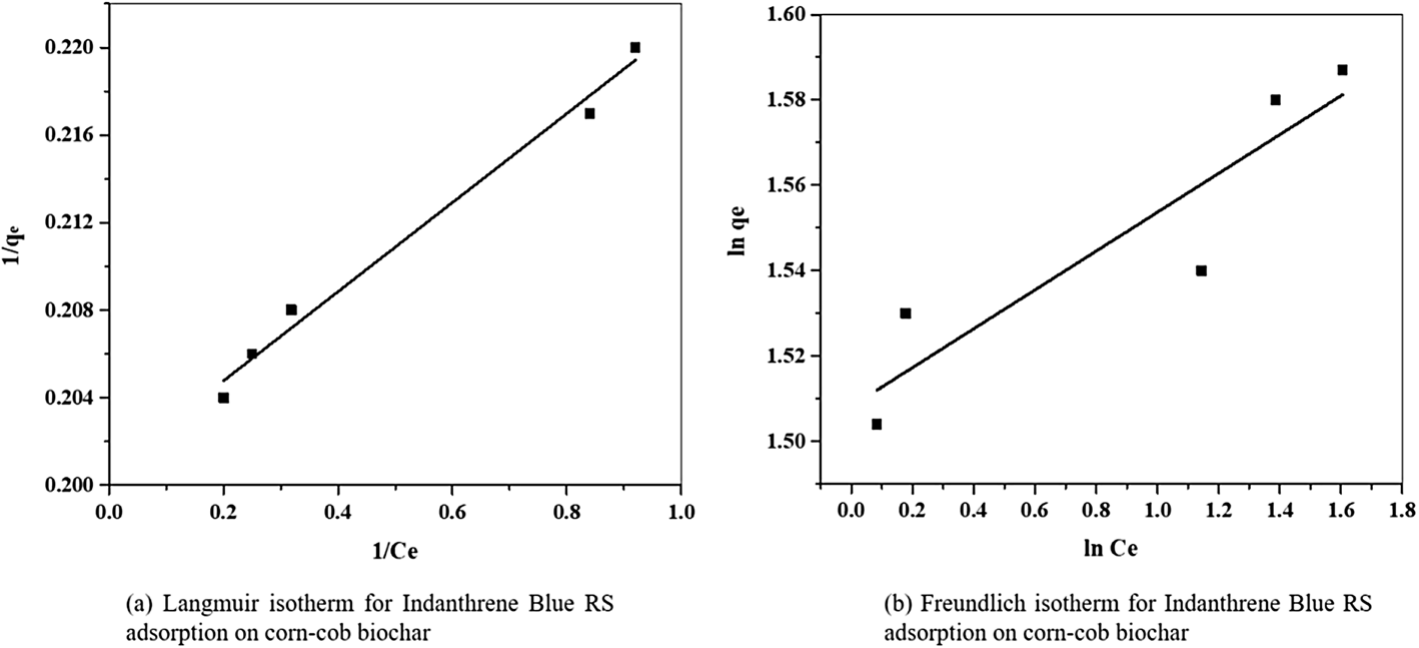
Isotherms for Indanthrene Blue RS adsorption.

The experimental equilibrium data were plotted (Fig. 4a,b) using the Langmuir and Freundlich isotherm model at 30 °C. The coefficient of correlation for Langmuir and Freundlich adsorption isotherm is determined by applying the experimental adsorption equilibrium results and is given in Table 2. The Langmuir isotherm correlation coefficient (R^2^ = 0.98) is higher than the Freundlich isotherm obtained value (R^2^ = 0.84).

**Table 2:** Isotherm constants for Indanthrene Blue RS adsorption onto corn-cob biochar.

Adsorbent	Langmuir constants				Freundlich constants		
	Q_0,__cal_ (mg g^−1^)	Q_0,__exp_ (mg g^−1^)	K_L_	R^2^	K_f_	N	R^2^
Corn-cob biochar	4.55	4.98	4.92	0.98	4.52	21.97	0.84

As the dye concentration increases, the adsorption capacity increases, suggesting that the available binding sites were saturated immediately as more dye was present in the aqueous solution^42^. In an aqueous solution, the dye molecule and the corn-cob biochar exist as cation and anion, respectively. Consequently, adsorption is considered the chemisorption in nature and describes that the adsorption is taking place on strongly homogeneous surfaces.

The equilibrium data for the Indanthrene Blue RS adsorption onto CC-biochar are best fitted with the Langmuir isotherm model, indicating that the monolayer coverage of the dye molecule occurs over the adsorbent surface. In the Freundlich isotherm model, the value for n greater than 1 represents the favorable adsorption, and the adsorbent is efficient for the entire range of dye concentrations^62^. Therefore, the Langmuir isotherm model would reflect the adsorption mechanism better physically than the Freundlich isotherm model (Table 2).

### Batch biodegradation

**Figure 5:**
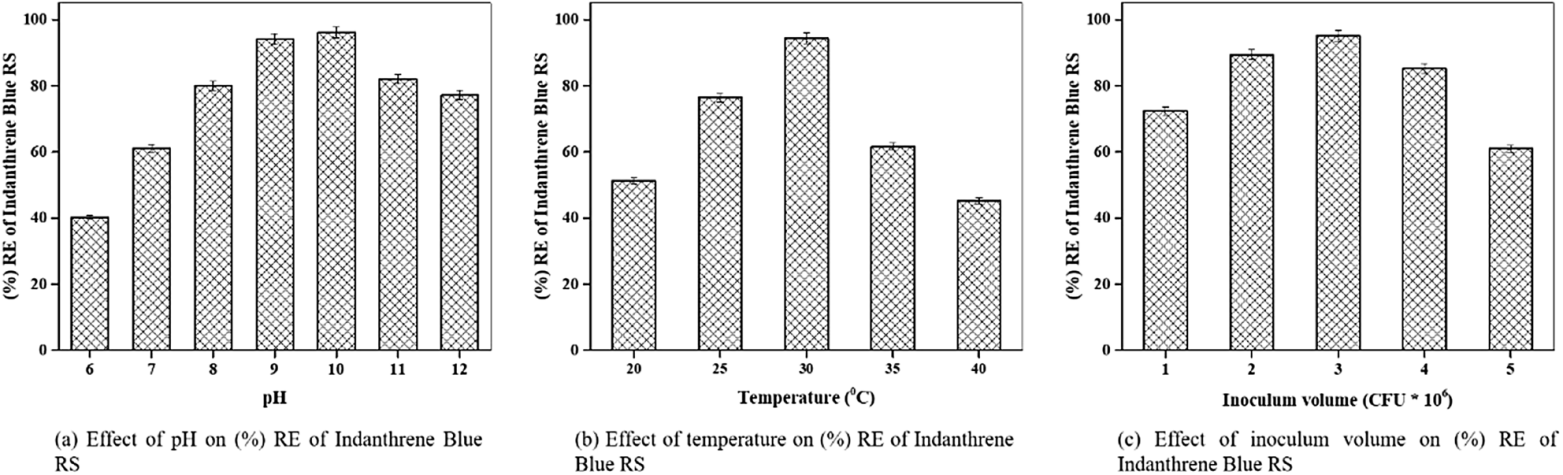
Parameter optimization in up-flow packed bed bioreactor.

Batch experiments were carried out by varying the optimum parameters such as pH, temperature, and inoculum volume to evaluate the optimal values for the Indanthrene Blue RS decolorization. It was observed that the removal percentage of Indanthrene Blue RS increases rapidly with a pH increases from 9.0 to 10.0 with an optimum pH of 10.0, at which the RE of 96.2% was found (Fig. 5a). The temperature range of 20 to 40 °C was evaluated. It was found that the RE increased at 30 °C to 94.3% and then decreased drastically (Fig. 5b), similar to the findings of Das et al.^63^. As the inoculum volume increased from 1.0 × 10^6^ to 5.0 × 10^6^ CFU mL^−1^, the RE increased rapidly to 95.2% at an inoculum volume of 3.0 × 10^6^ CFU mL^−1^ (Fig. 5c). The optimal value of pH, temperature, and inoculum volume were found to be 10.0, 30 °C, and 3.0 × 10^6^ CFU mL^−1^, respectively.

### Biodecolorization of Indanthrene Blue RS in continuous up-flow packed bed bioreactor

UFPBBR was performed within the concentration range of 100 to 500 mg L^−1^ to assess the impact of the initial dye concentration on removal percentage using optimal parameters achieved from batch experiments. The percentage removal of Indanthrene Blue RS at an initial dye concentration of 500 mg L^−1^ increases by about 90%. It decreases with a subsequent rise in dye concentration owing to the inhibitory effects of substrates. Supplementary Fig. 5 illustrates the effect of different initial Indanthrene Blue RS concentrations on the adsorption capacity of CC-Biochar and bacteria-immobilized CC-Biochar. It was, therefore, evident that the immobilization significantly improved the ability of the microbial cells to degrade the dye effectively.

The increased surface area of CC-biochar enabled the interactions between the dye molecules and bacterial cells at higher concentrations to achieve a higher decolorization percentage. The dye molecules were adsorbed by the pores and the active sites found on the surface of the CC-biochar and thus established active target sites for the immobilization of the bacterial cells. The active sites on the CC-biochar transform the dye molecules into environmentally friendly by-products with the help of the bacteria immobilized on the biochar and thus renew the porous structure further for adsorption^64,65^. A synergistic effect was established by the subsequent degradation of Indanthrene Blue RS dye by the bacterial cells together with active sites on the surface of the biochar that attributed to the significant dye degradation at higher concentrations. Similar findings have been documented in earlier studies showing the effectiveness of immobilized cell systems for dye degradation^30,66^.

#### Effect of flow rate on removal efficiency

**Figure 6:**
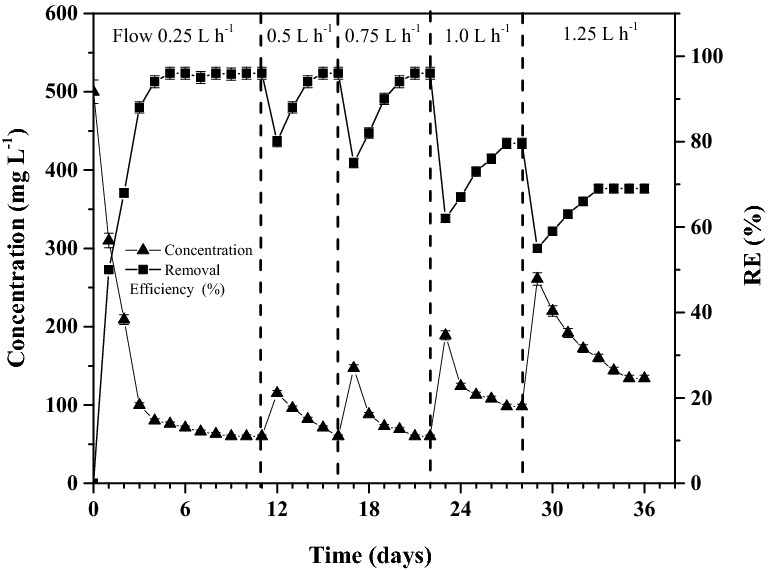
Bioreactor performance with changing inlet Indanthrene Blue RS feed flow rate.

The UFPBBR efficiency was evaluated in continuous mode (CUFPBBR) using optimal parameters acquired from batch experiments by differing the feed flow rate (0.25, 0.5, 0.75, 1.0, and 1.25 L h^−1^) Indanthrene Blue RS concentration in the range as in UFPBBR. In a continuous process, the flow rate determines the dye's decolorization within the effluent. Initially, the bioreactor was maintained at the feed flow rate of 0.25 L h^−1^ to enable adequate bacterial growth, with glucose as a carbon source, and to maintain a steady-state condition. On the 6^th^ day of operation, the steady-state was established, which is apparent with the almost constant removal efficiency (95%) (Fig. 6). It was observed that about 6 days are required to acclimate consortium-BP to decolorize Indanthrene Blue RS effectively.

Moreover, a significant reduction in decolorization was reported at high flow rates. The flow rate was increased on the 11th day to 0.50 L h^−1^. On the 12th day, a rapid decrease in RE was found the following, which the RE amended and then became stable at 95% on the 15th day. The flow rates were improved to 0.75 L h^−1^ on the 16th day. The bioreactor performance was similar, a significant decline led by a 95% recovery in removal efficiency and stabilization. On the 22nd day, the flow rate increased to 1.0 L h^−1^, and after a sudden decline, the RE stabilized at 79.6%. The steady-state RE value dropped significantly at a flow rate of 1.25 L h^−1^ and maintained at about 69%.

The decline in RE value was sharper above the flow rate of 0.75 L h^−1^, indicating a change in the bioreactor’s control mechanism^56^. The interaction of the dye molecule with cells immobilized on CC-biochar requires a longer retention time. Chen et al. 2005 reported that the dye molecules required more retention time to interact with the bacteria cell than the time necessary for the intraparticle diffusion^67^. Though biosorption took place during the process, and there is a possibility that the consortia cells used the adsorbed dye onto CC-biochar for degradation. It was observed that nearly 6% to 8% of the decolorization was due to the biosorption of the dye onto the biochar, with most of the decolorization attributed to the dye utilization by the bacterial consortia^68^. Biodegradation is attributed to the dye degradation process in the present work since the adsorption process only occurs during the initial stage of reactor operation. The continuous BB process can be divided into two phases. In the first phase, the concentration of Indanthrene Blue RS gradually decreases, suggesting that biosorption on CC-biochar plays a leading role and that much of the Indanthrene Blue RS molecules are adsorbed. The high adsorption capacity of CC-Biochar thus eliminates the toxic risk to the immobilized bacterial cells. CC-biochar reaches the highest adsorption value at equilibrium in the second phase, and biodegradation occurs considerably until the Indanthrene Blue RS is eliminated. Throughout the entire continuous BB process, the continuous removal of Indanthrene Blue RS significantly differs from the usual CC-Biochar adsorption process.

#### Effect of Indanthrene Blue RS inlet loading rate on elimination capacity and removal efficiency

**Figure 7:**
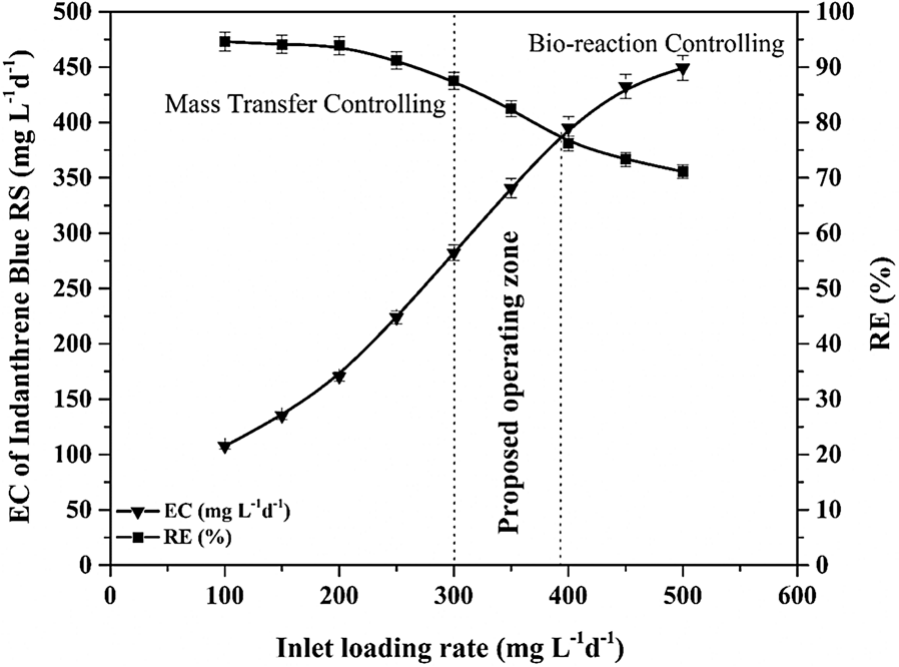
Effect of inlet Indanthrene Blue RS load on elimination capacity and removal efficiency.

The difference in the removal efficiency and elimination capacity of the Indanthrene Blue RS at varying inlet loading rates is illustrated in Fig. 7. The Indanthrene Blue RS inlet loading plot displays two separate regions for the mass transfer zone and bio-reaction zone. With an increment in the inlet loading rate of Indanthrene Blue RS from 100 to 500 mg L^−1^ day^−1^, the RE was continuously increasing above 90% to the loading rate of 300 mg L^−1^ day^−1^, after which it continually decreased. EC linearly increased with an increasing inlet loading rate of Indanthrene Blue RS and reached its highest value of 446.1 mg L^−1^ day^−1^ at the loading rate of 500 mg L^−1^ day^−1^. Similarly, the EC keeps increasing with an increase in Indanthrene Blue RS loading, which is evident from a slight variation in the EC vs. Inlet loading rate plot (Fig. 7). Such results thus confirm the difference in the reactor controlling mechanism throughout the entire biodegradation process^56,69,70^.

The diffusional flux through the biofilm would be reduced at low loading rates, resulted in mass transfer limitations. The inner part of the biofilm at the packing media surface remains substrate deficient and is never used by microbes for effective biodegradation. Whereas high loading rates resulted in higher diffusional flux, thereby changing the process between the mass transfer zone to the bio-reaction controlling zone. The contaminant rapidly approaches the innermost layers of biofilm, and microbes use the adequate substrate available through the biodegradation control mechanism. At the initial loading rate of 300 mg L^−1^ day^−1^, it was observed that the process moved from the mass-transfer zone to the bio-reaction controlling region (Fig. 7).

A rise in the inlet loading rate from 100 to 300 mg L^−1^ day^−1^ resulted in increased use of the biofilm corresponding to nearly constant RE with a significant increase in EC value. The substrate inhibition with higher concentrations of Indanthrene Blue RS may lead to decreased RE^56^. The operation of bioreactors with an appropriate value of RE in the bio-reaction controlling region is always satisfactory. The optimal operating range for this study lies around 300 to 395.4 mg L^−1^ day^−1^. The inlet loading rate where the process in the bioreactor moves from a mass transfer zone to the bio-reaction controlling zone and the RE and EC plot intersection value can be used for industrial applications as an estimated calculation of the bioreactor’s operating concentration range (Fig. 7). Therefore, the continuous immobilized UFPBBR showed promising results that can be used for practical applications.

## Conclusion

The UFPBBR immobilized with CC-biochar significantly increased the ability of the bacterial cells to degrade the dye effectively. The increased surface area of CC-biochar enabled the interactions between the dye molecules and bacterial cells to achieve a higher decolorization percentage at higher concentrations. The adsorption kinetics showed that the Indanthrene Blue RS adsorption on CC-biochar followed the pseudo-second-order kinetic model. Similarly, the adsorption of Indanthrene Blue RS on CC-biochar followed the Langmuir isotherm model with the maximum adsorption capacity of 4.55 mg g^−1^ at 30 °C. An increase in the inlet loading rate of Indanthrene Blue RS from 100 to 500 mg L^−1^ day^−1^, the RE is continually increasing above 90% to the loading rate of 300 mg L^−1^ day^−1^, after which; it begins to decrease. The EC value continued to increase linearly with Indanthrene Blue RS inlet loading rate and reached the highest value of 446.1 mg L^−1^ day^−1^ with RE of 75.2% at 500 mg L^−1^ day^−1^ loading rate. The present study combined the biosorption and biodegradation processes to develop an efficient treatment strategy. The above results suggest that the developed consortium-BP and the continuous immobilized UFPBBR have shown promising results that combine the advantages of both biosorption and biodegradation processes. The UFPBBR can be used for practical applications at an industrial scale in decolorizing and simultaneously reducing the toxicity of textile effluents containing anthraquinone dyes.

## Supplementary Information

Supplementary Information.

## Data Availability

Data are, however, available from the authors upon reasonable request and with permission.
