# Supplementary material for: Biodegradation of Indanthrene Blue RS dye in immobilized continuous upflow packed bed bioreactor using corncob biochar
Source: Sci Rep. 2021 Jun 28;11:13390. doi: 10.1038/s41598-021-92889-3 (PMC8238989; doi:10.1038/s41598-021-92889-3)
Supplement: Supplementary file 1 — Supplementary Information. [file 41598_2021_92889_MOESM1_ESM.pdf]

Biodegradation of Indanthrene Blue RS dye in immobilized continuous upflow packed bed bioreactor using corncob biochar

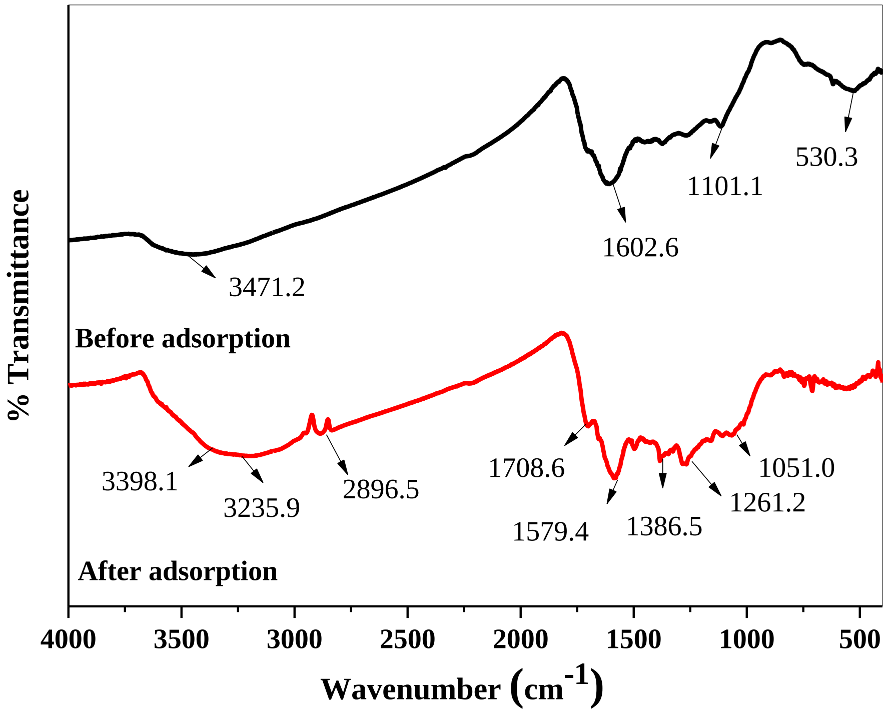

Supplementary Figure 1: FT-IR results of corn-cob biochar before and after sorption.

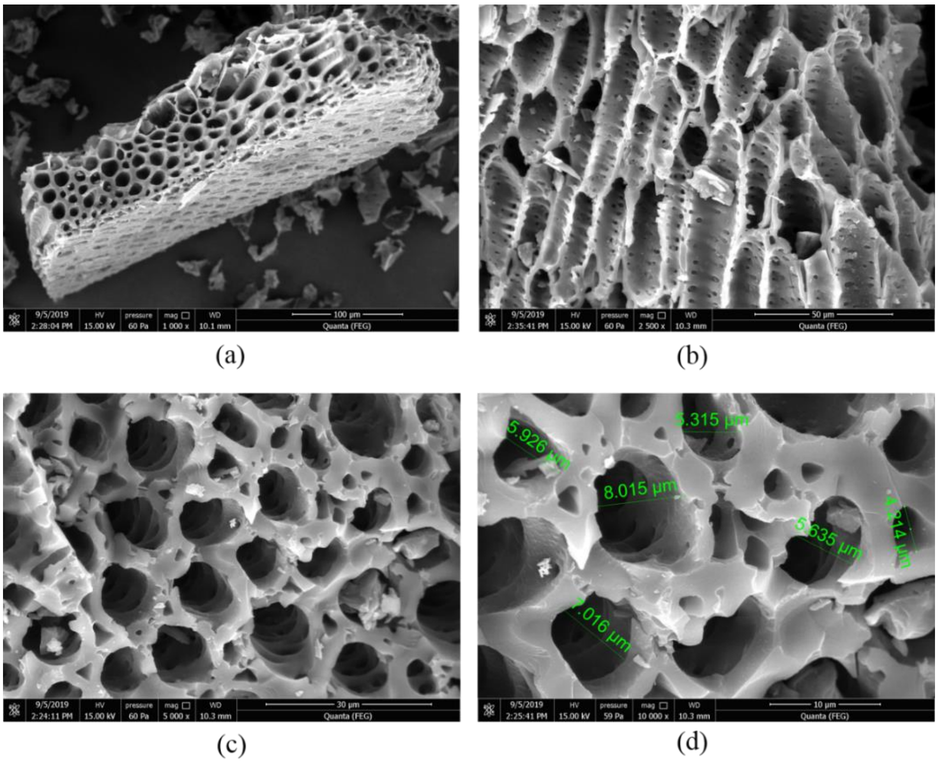

Supplementary Figure 2: SEM image for surface morphology (pore space) of the corn-cob biochar (a) at 1000x (b) at 2500x (c) at 5000x (d) at 10000x.

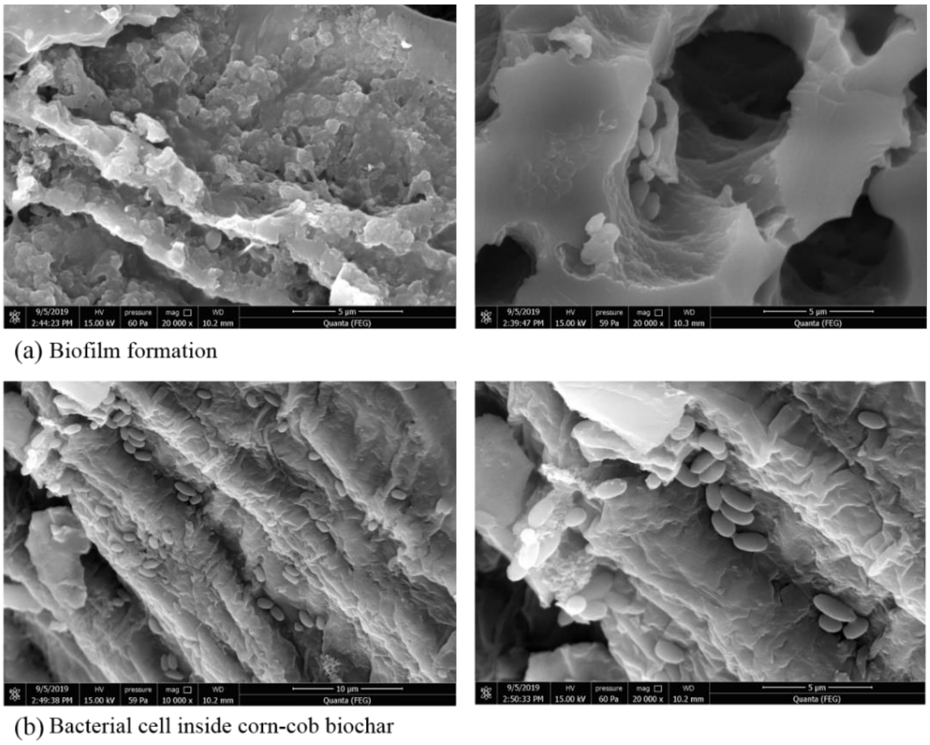

Supplementary Figure 3: (a) Biofilm formation on the corn-cob biochar surface, (b) penetration of the bacteria cells inside the corn-cob biochar.

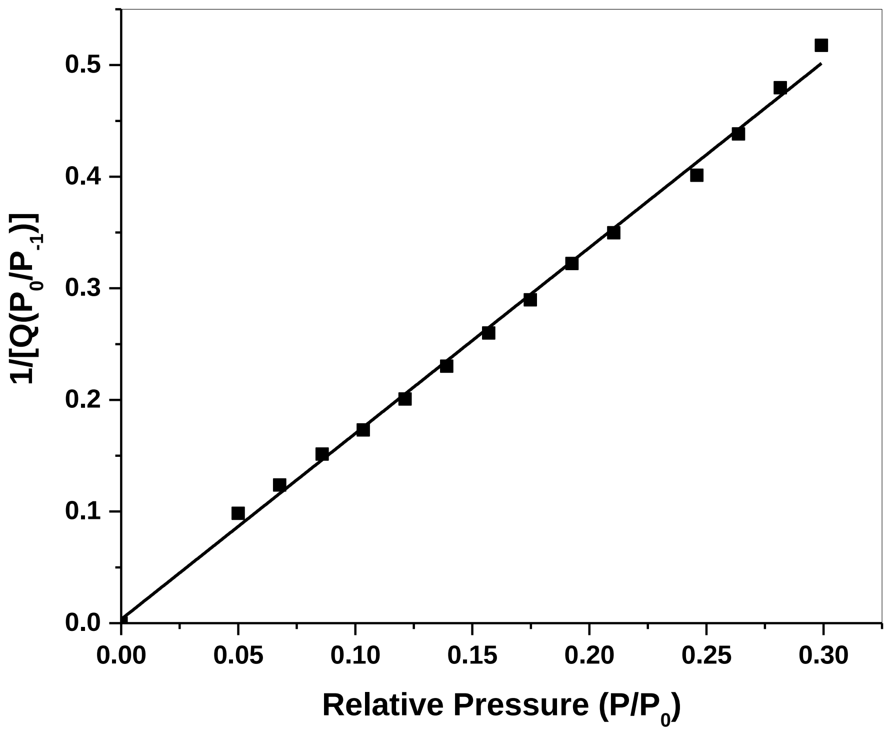

Supplementary Figure 4: BET surface area plot for the corn-cob biochar.

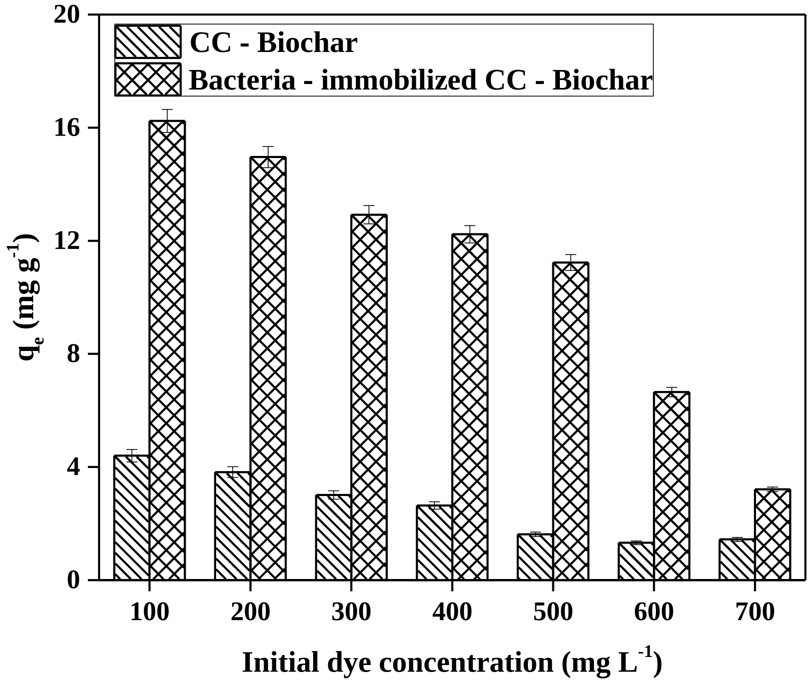

Supplementary Figure 5: Effect of different initial Indanthrene Blue RS concentration on the adsorption capacity of CC-Biochar and Bacteria-immobilized CC-Biochar.

Supplementary Table 1: CHNS analysis of corn-cob biochar before and after sorption.

Element	Before Sorption (%)	After Sorption (%)
C	78.78	70.97
H	1.13	1.22
N	10.29	14.96
S	0.55	0.24
